# Replacement of TCR Dβ With Immunoglobulin D_H_ DSP2.3 Imposes a Tyrosine-Enriched TCR Repertoire and Adversely Affects T Cell Development

**DOI:** 10.3389/fimmu.2020.573413

**Published:** 2020-09-29

**Authors:** Michael Levinson, Mohamed Khass, Peter D. Burrows, Harry W. Schroeder

**Affiliations:** ^1^Division of Clinical Immunology and Rheumatology, Department of Medicine, University of Alabama at Birmingham, Birmingham, AL, United States; ^2^Division of Genetic Engineering and Biotechnology, National Research Center, Cairo, Egypt; ^3^Department of Microbiology, University of Alabama at Birmingham, Birmingham, AL, United States

**Keywords:** germline selection, CDR-B3, TCRB diversity, Db1, immunoglobulin DSP2.3

## Abstract

Enrichment for tyrosine in immunoglobulin CDR-H3 is due in large part to natural selection of germline immunoglobulin D_H_ sequence. We have previously shown that when D_H_ sequence is modified to reduce the contribution of tyrosine codons, epitope recognition is altered and B cell development, antibody production, autoantibody production, and morbidity and mortality following pathogen challenge are adversely affected. TCRβ diversity (Dβ) gene segment sequences are even more highly conserved than D_H_, with trout Dβ1 identical to human and mouse Dβ1. We hypothesized that natural selection of Dβ sequence also shapes CDR-B3 diversity and influences T cell development and T cell function. To test this, we used a mouse strain that lacked Dβ2 and contained a novel Dβ1 allele (*DβYTL*) that replaces Dβ1 with an immunoglobulin D_H_, DSP2.3. Unlike Dβ1, wherein glycine predominates in all three reading frames (RFs), in DSP2.3 there is enrichment for tyrosine in RF1, threonine in RF2, and leucine in RF3. Mature T cells using *DβYTL* expressed TCRs enriched at particular CDR-B3 positions for tyrosine but depleted of leucine. Changing Dβ sequence altered thymocyte and peripheral T cell numbers and the T cell response to an ovalbumin immunodominant epitope. The differences in tyrosine content might explain, at least in part, why TCRs are more polyspecific and of lower affinity for their cognate antigens than their immunoglobulin counterparts.

## Introduction

During T and B cell development, V(D)J rearrangement and N addition have the potential to create more than 10^16^ different T cell receptor (TCR) or immunoglobulin (Ig) antigen binding sites (1, 2). However, with lymphocytes in the body numbering 10^9^ or fewer cells, only a small fraction of this diversity is accessible at any given time. Lymphocytes bearing antigen receptors that will properly recognize pathogens need to be available at the time of antigen challenge (3, 4), and their absence can create susceptibility to infection. Conversely, a broad antigen receptor repertoire will include autoreactive clones that need to be minimized to avoid autoimmune disease. These observations highlight some of the pressures under which the adaptive immune system labors to balance competing demands for diversity, protection and efficiency within finite constraints on lymphocyte numbers and time.

One method to achieve a balance between these demands would be to program the system to produce preimmune repertoires that facilitate lymphocyte development and function, i.e., to postulate the existence of predetermined, “preferred,” or even locally “optimal” preimmune repertoires. To test this hypothesis, we previously scrutinized Ig repertoires from developing B lineage cells and found evidence of consistent preferences in the amino acid composition of the antigen binding site (5). We have shown that these preferences reflect natural selection of D_H_ sequence followed by subsequent somatic selection when the cells pass through developmental checkpoints (6). In this work, we sought to test whether similar selective processes were influencing Dβ germline sequence and, ultimately, T cell receptor diversity, development and function.

Complementarity determining region 3 (CDR3) is the direct product of V(D)J rearrangement (7). Within the Ig and TCR variable domains, CDR3 has been shown to be the focus of preimmune repertoire diversification. Paired CDR3s belonging to the two chains that create the antigen receptor lie at the center of the antigen binding site where they often play a determinant role in the antigen binding properties of that receptor, and thus in the fate and function of the cell that bears it (7–9). In TCRαβ, the CDR3s are of special importance because they are typically positioned to interact directly with the peptide displayed by an MHC molecule (10).

During VDJ rearrangement, the somatic introduction of amino acids at random by the TdT-catalyzed addition of non-germline-encoded N nucleotides in CDR3 provides a means to free Ig and TCR repertoires from germline encoded amino acid constraints (7). The inclusion of a D, for diversity, gene segment allows two rounds of N diversification, making Ig heavy (H) and TCR β chain CDR3s more diverse than their Ig light (L) and TCRα partners.

In B cells, restrictions in CDR-H3 diversity begin with natural selection of D_H_ gene sequence. Individual D_H_ reading frames exhibit evolutionarily conserved amino acid signatures (11). Coupled with reading frame preferences, the sequence of the D_H_ promotes general enrichment in CDR-H3 for tyrosine and against hydrophobic amino acids (e.g., leucine and valine) (5). After VDJ rearrangement is complete, surrogate light chain imposes somatic selective pressures, further enriching for tyrosine and against hydrophobic amino acids at specific positions within CDR-H3 (12). Alterations in the D_H_ sequence result in D sequence-specific changes to the CDR-H3 repertoire that cannot be fully corrected by preBCR selection. Such alterations in the CDR-H3 repertoire can lead to diminished numbers of peripheral B cell subsets, reductions in antibody production, altered patterns of epitope recognition, impaired affinity maturation, increased morbidity and mortality following pathogen challenge, and an increased likelihood of generating self-reactive antibodies (5). Here we report evidence of an alternative set of amino acid preferences in TCRβ CDR-B3 that are also imposed by natural selection of Dβ sequence and, if modified, also do not fully correct after somatic selection during passage through T cell developmental checkpoints. As in the case of D_H_ sequence in B cells, when these restrictions placed by natural selection of germline Dβ gene segment sequence are violated, peripheral T cell numbers and function are altered.

## Materials and Methods

### Phylogenetic Trees

Phylogenetic trees were created using the Interactive Tree of Life (iTOL) at itol.embl.de (13).

### Dβ2KO and DβYTL Mice

We studied TCR repertoire and T cell development in two C57BL/6 mouse strains with altered Dβ sequence (14), as well as in normal C57BL/6 controls. The first strain contained a deletion of the Dβ2-Jβ2 locus (15, 16) and is termed Dβ2KO. In the second strain, we replaced the Dβ1 gene segment in the Dβ2KO with DSP2.3, a commonly used Ig D_H_ gene segment, to create a new TCRβ allele. We termed this new D_H_ substituted TCR locus DβYTL, which refers to the central amino acids in each of its three translated reading frames, i.e., tyrosine, threonine and leucine. All animal experiments were approved by the University of Alabama at Birmingham (UAB) Institutional Animal Care and Use Committee. The UAB Animal Care and Use program is fully accredited by Association for Assessment and Accreditation of Laboratory Animal Care Committee.

### Flow Cytometric Analysis and Cell Sorting

Single cell suspensions were prepared from both the thymus and spleen of two mice. Red blood cells were removed with RBC lysing solution (1 mM KHCO_3_, 0.15 M NH_4_Cl, and 0.1 mM Na_2_EDTA). Cells were washed twice and resuspended in a master-mix of staining buffer containing optimal concentrations of monoclonal antibody reagents. Samples were analyzed on a FACS LSR II (Becton Dickinson) or sorted with a FACS Aria (Becton Dickinson). Double negative thymic cells were stained with PE-Cy7- CD25 (BD Cat#552880), APC- CD44 (BD Cat#559250), biotinylated- CD28 (BD Cat#553296) (developed secondarily with streptavidin), and a lineage stain [PE-CD3 (BD Cat#555275), monoclonal PE-CD4 (BD Cat#553049), PE-CD8α (BD Cat#553033), PE-B220 (BD Cat#561878), PE-CD11b (BD Cat#553311), PE-NK1.1 (BD Cat#553165)] to remove mature T, B, and NK cells. Double and single positive thymic cells were stained with PE-CD3 (BD Cat#555275), FITC-CD4 (BD Cat#557307), and APC-CD8α (BD Cat#557682). Splenic cells were stained with PE-CD3 (BD Cat#555275), FITC-CD4 (BD Cat#557307), and APC-CD8α (BD Cat#557682). All subsets were also stained with propidium iodide (PI) to exclude dead cells.

### RNA, RT-PCR, Cloning, and Sequencing

Total RNA was prepared from 1 to 2 × 10^4^ cells of each individual subset, sorted directly into RLT lysing buffer using the QIAGEN RNeasy mini-kit. RNA was used to synthesize cDNA using the QIAGEN RT-PCR Kit and the manufacturer’s recommended protocol under the following conditions: 95°C denaturation for 2 min; 30 cycles of 94°C for 1 min, 60°C for 1 min, and 72°C for 1 min; and a final 72°C extension for 10 min. The reaction buffer contained 100 mM Tris–HCl, pH 8.8, 15 mM MgCl_2_, and 750 mM KCl. Primers used were TCRB13-1 (5’- TGCTGGCAACCTTCGAATAGGA-3’) and TCRBC1 (5’- TGAGAAATGTGACTCCACCCA-3’). PCR products were cloned (TOPO-TA Cloning Kit; Invitrogen) and sequenced using the primer TCRB13-1 on an ABI 3730 sequencer.

### Sequence Analysis of CDR-β3

Gene segments were assigned according to published germline sequences for the TCR β gene segments as listed in the ImMunoGeneTics database (17). The CDR3 of the TCRβ chain was defined to include those residues located between the conserved cysteine (C104) of FR3 and the conserved phenylalanine (F118) of FR4 (18).

### Sequence Logos

Sequence logos were created using R with the motifStack package from Bioconductor (19).

### Immunizations

Mice 8–12 weeks of age were immunized intraperitoneally with 100 μg of ovalbumin protein. Spleens were removed 7 days later and stained with monoclonal APC-CD8α (BD Cat#557682) and PE-OVA(257–264) peptide-H-2K(b) (NIH Tetramer Core) and analyzed on a FACS LSR II (Becton Dickinson).

### Statistical Analysis

Statistical analysis was performed with JMP version 13.1.0 (SAS Institute). Population means were analyzed using an unpaired two-tailed Student’s *t*-test or an analysis of variance (ANOVA) as appropriate. For frequencies of individual amino acids, Pearson’s *χ*^2^ test was performed.

## Results

### The Sequence of Dβ1 Is Highly Conserved Among Jawed Vertebrates

**FIGURE 1:**
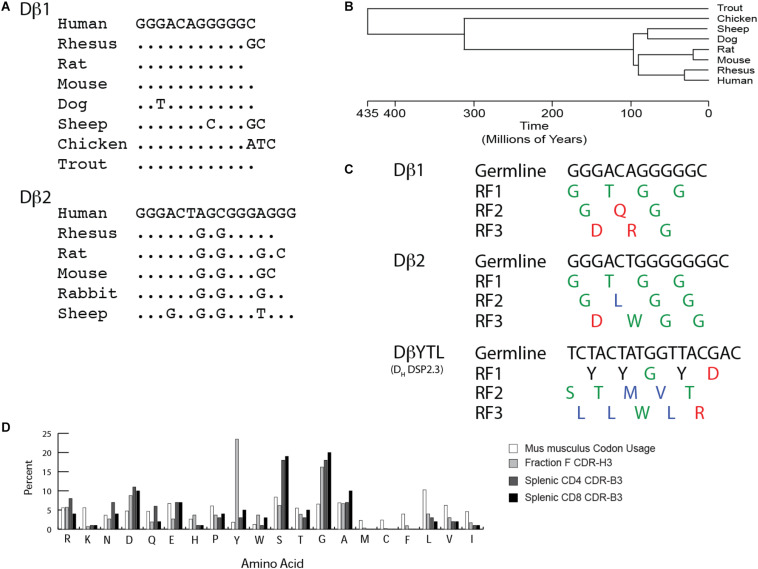
TCR Dβ gene segments are highly conserved. As with immunoglobulin D_H_, they exhibit distinct biases toward particular amino acids. **(A)** Conservation of Dβ sequence across jawed vertebrate species. **(B)** Timeline of divergence of select jawed vertebrate subgroups based on evolution. **(C)** Germline sequence and amino acid translation of mouse Dβ1, Dβ2 and mutant DβYTL. Single letter amino acids are color coded: red, charged amino acids; green, neutral amino acids; blue, hydrophobic amino acids, and tyrosine is in black. **(D)** Comparison of baseline murine codon usage to amino acid usage in mature splenic CD4 and CD8 T cells, and in mature, recirculating (Fraction F), bone marrow B cells in mice. Amino acids are listed from the most charged (left) to the most hydrophobic (right) per the Kyte-Doolittle scale as modified by Eisenberg (38, 39).

The genomic coding sequences of trout, mouse and human Dβ1 are base for base identical (Figure 1A). Conservation of Dβ1 across more than 400 million years of evolution suggests that its sequence is under intense natural selection (Figure 1B). Species that contain a Dβ2 demonstrate similar sequence conservation for this Dβ. Unlike preB cells, thymocytes do not evidence a D reading frame bias. Of the 22 amino acids encoded by Dβ1 and Dβ2 in mice, there are 17 neutral amino acids. Of these, glycine contributes 14, threonine two, and tryptophan one. In addition, there are four charged amino acids encoded by Dβ1, including two aspartic acids, one glutamine, and one arginine; but only one hydrophobic amino acid, leucine (Figure 1C). Conversely, the three RFs in a typical mouse D_H_ have very distinct amino acid signatures, with one RF enriched for tyrosine and two enriched for hydrophobic amino acids, especially leucine in RF3. As in the case of CDR-H3, the percentage of global amino acid content in CDR-B3 from mature T cells correlates with the distribution of amino acids encoded by Dβ (Figure 1D), with an overabundance of glycine and aspartic acid.

**FIGURE 2:**
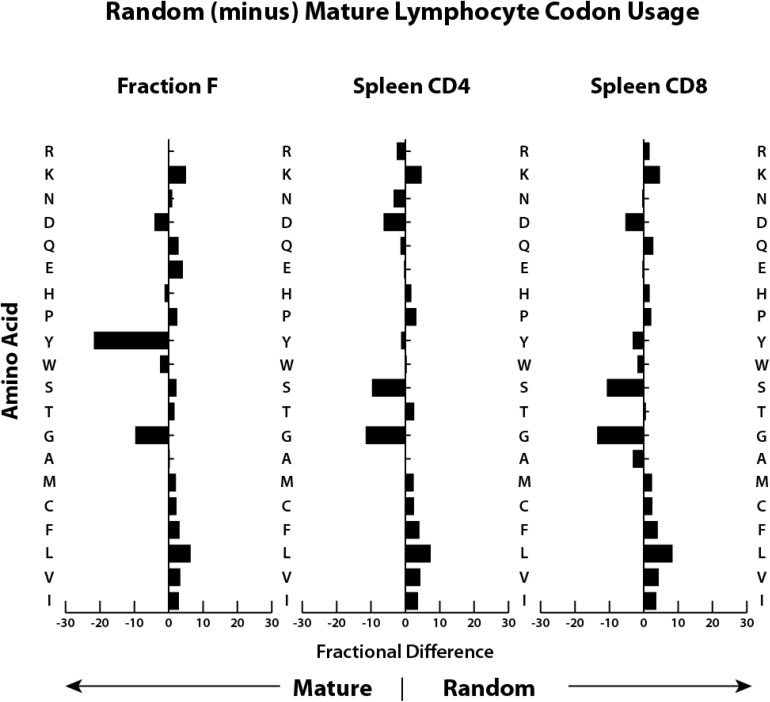
Both T cell CDR-B3 and B cell CDR-H3 demonstrate global biases in the usage of particular amino acids. Shown is the fractional difference between that amino acid’s frequency of occurrence (in percent) in GenBank protein sequences for mice (11) and the frequency of amino acids present in CDR3 sequence from immunoglobulin H chains from mature B cells (Fraction F) from the bone marrow (40) and from TCRβ cloned from splenic CD4 and CD8 T cells (this work).

A comparison of fractional differences in amino acid utilization between bone marrow mature (Hardy Fraction F) B cells and splenic CD4 and CD8 T cells emphasizes that the most prominent differences between CDR-H3 and CDR-B3 sequences is the excess of tyrosine in CDR-H3 versus a greater preference for glycine in CDR-B3 (Figure 2). These observations led us to the hypothesis that, as in the case of D_H_, the sequence of Dβ is used to bias the amino acid content of CDR-B3.

### Replacement of Dβ1 With D_H_ DSP2.3 Yields a Preimmune TCR CDR- B3 Repertoire Enriched for Tyrosine, Threonine, and Leucine

Previously, in order to test the role of germline D sequence on the outcome of VDJ rearrangement in early thymocytes, we created a (DβYTL) mouse with a mutant TCRβ DJC locus wherein Dβ2 and Jβ2 gene segments had been deleted (Dβ2KO) and the remaining Dβ1 gene segment [ImMunoGeneTics (IMGT) database (5) (IMGT: TRBD1^∗^01)] replaced with a commonly used D_H_ gene segment DSP2.3 [IMGT: IGHD2-7^∗^01 (BALB/c)] (14). DSP2.3 is a frequently used mouse D_H_, and thus represents a fully functional diversity gene segment that has undergone natural selection in the immunoglobulin locus.

We chose DSP2.3 because, unlike most D_H_ but like Dβ1 and Dβ2, it does not include a termination codon in reading frame 3. We termed the altered Dβ locus DβYTL, which emphasizes the most common amino acid in each of the three reading frames. In addition to allowing testing of the role of Dβ sequence on the pre-immune CDR-B3 amino acid repertoire in general, the DβYTL allele allows competition between T cells bearing CDR-B3 enriched for tyrosine, threonine, or leucine within the same mouse and without further manipulation since all three reading frames are equally accessible to rearrangement (Figure 1C). The TCRβ allele lacking the Dβ2-Jβ2 locus was termed Dβ2KO. Although the deletion of the Dβ2-Jβ2 locus is artificial, the Dβ2KO allele mirrors the physiologic absence of Dβ2-Jβ2 observed in NZW mice (12).

Alteration of the Dβ locus in DβYTL with preservation of Vβ permits creation of a polyclonal repertoire differing from its Dβ2KO control only in Dβ coding sequence, and from WT in both Dβ sequence and the loss of Jβ2. These Dβ2KO mice were used to control for the deletion of the Dβ2-Jβ2 gene segment cluster in the DβYTL allele. Initial targeting was performed in a 129/Sv ES cell line. Chimeric C57BL/6 mice were crossed for 24 generations onto C57BL/6. The integrity of the Dβ1 locus was confirmed by sequencing.

### Replacement of Dβ1 With D_H_ DSP2.3 Leads to a Reduction in Peripheral T Cell Numbers

**FIGURE 3:**
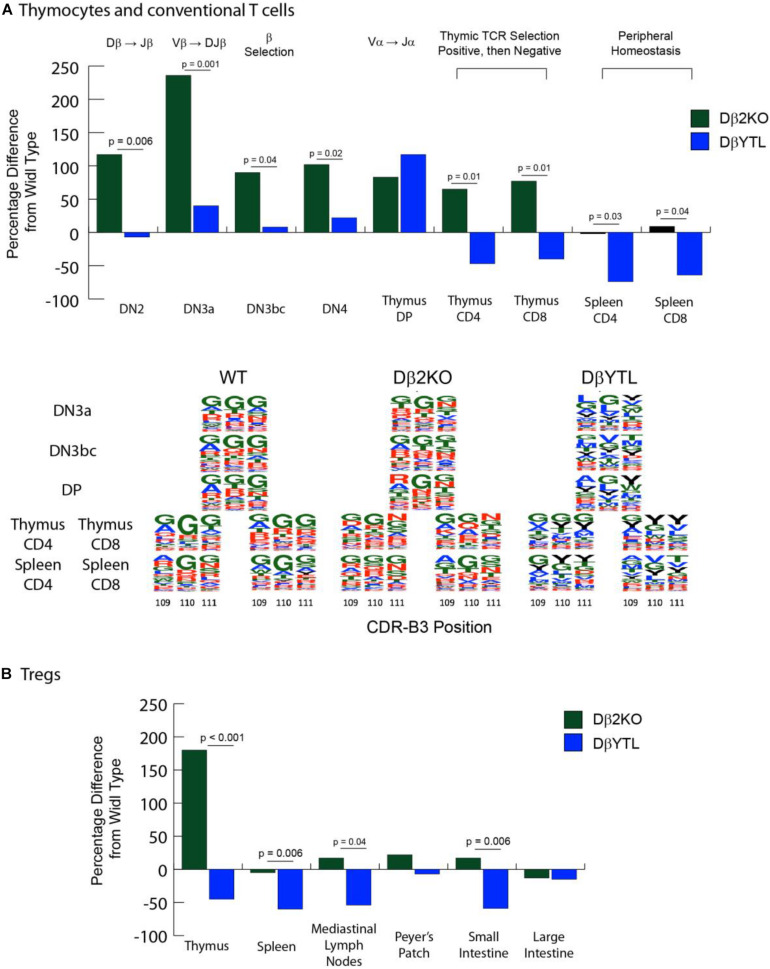
Changes in cell numbers during T cell development and in the amino acid composition of the CDR-B3 repertoire in the different mouse strains. [**(A)**, top] Compared to WT, the percent loss or gain in absolute numbers of Dβ2KO and DβYTL thymocytes and conventional T cells during development. Double negative (DN) thymocytes lack CD4 or CD8 expression. DβJβ rearrangement begins at the DN2 stage, followed by Vβ DJβ rearrangement in DN3a. Testing for a productive TCR Vβ chain (β selection) occurs in DN3bc. VαJα occurs in DN4. CD4/CD8 double positive (DP) thymocytes express complete TCR αβ. MHC-peptide then selects for a single co-receptor in the thymus (either thymus CD4 and CD8) before the nascent T cells migrate to the periphery (splenic CD4 or CD8). Above the figure is shown either V(D)J re-arrangement steps or somatic selection mechanisms. In the thymus at the single positive step, positive selection checks whether the TCR can bind to MHC-peptide, and negative selection deletes clones that are highly self-reactive. Data is from five individual mice, repeated twice. [**(A)**, bottom] Sequence logos of CDR3 positions 109, 110, and 111 of WT, Dβ2KO, and DβYTL through selected development stages. Single letter amino acids are color coded: red, charged amino acids; green, neutral amino acids; blue, hydrophobic amino acids, and tyrosine, black. **(B)** Compared to WT, the percent loss or gain in absolute numbers of Dβ2KO and DβYTL Foxp3 + Treg cells in the thymus, spleen, and selected gut tissues. For both **(A,B)**, the *p*-values that reached statistical significance are shown for the comparison between Dβ2KO and DβYTL cell numbers.

We used flow cytometry to quantify and sort T lineage cells marked by their role in key TCR quality control checkpoints in the thymus and spleen (Supplementary Figure 1). WT, homozygous Dβ2KO, and homozygous DβYTL were bred and their offspring sacrificed at 8 weeks of age. The absolute numbers of double negative (DN2), DN3a, DN3bc, DN4, double positive (DP), CD4 and CD8 thymocytes, and splenic CD4 and CD8 T cells were determined. Table 1 reports the mean and the standard error of the mean for the absolute numbers from each T lineage subset. The percent increase or decrease in the mean for each individual T lineage cell subset as compared to WT is depicted in Figure 3A. A description of the checkpoints these subsets represent is given in the figure.

**TABLE 1:** Total numbers, SEM, and statistical analysis for T cell numbers through development in WT, Dβ2KO, and DβYTL.

**T lineage subset**	**WT ± SEM**	**Dβ2KO ± SEM**	**DβYTL ± SEM**	**WT vs. Dβ2KO**	**WT vs. DβYTL**	**Dβ2KO vs. DβYTL**
DN2	6.72E + 05 ± 1.65E + 05	1.46E + 06 ± 3.40E + 05	6.25E + 05 ± 9.16E + 04	0.006	0.61	0.006
DN3a	5.84E + 05 ± 1.28E + 05	1.96E + 06 ± 2.86E + 05	8.15E + 05 ± 7.52E + 04	<0.0001	0.26	0.001
DN3bc	3.54E + 05 ± 8.16E + 04	6.73E + 05 ± 1.47E + 05	3.83E + 05 ± 4.41E + 04	0.03	0.7	0.04
DN4	2.54E + 06 ± 7.44E + 05	5.13E + 06 ± 1.09E + 06	3.11E + 06 ± 5.30E + 05	0.06	0.42	0.02
DP	3.98E + 06 ± 7.06E + 05	7.30E + 06 ± 7.46E + 05	8.65E + 06 ± 2.09E + 06	0.01	0.03	0.5
Thymus CD4	6.90E + 06 ± 2.04E + 06	1.14E + 07 ± 2.80E + 06	3.68E + 06 ± 8.64E + 05	0.21	0.24	0.01
Thymus CD8	2.47E + 06 ± 4.02E + 05	4.37E + 06 ± 3.75E + 05	1.47E + 06 ± 2.93E + 05	0.01	0.09	0.01
Spleen CD4	1.93E + 07 ± 4.59E + 06	1.89E + 07 ± 5.18E + 06	4.96E + 06 ± 1.93E + 06	0.96	0.03	0.03
Spleen CD8	1.45E + 07 ± 3.48E + 06	1.58E + 07 ± 4.54E + 06	5.27E + 06 ± 2.23E + 06	0.82	0.04	0.04

Deletion of the Dβ2-Jβ2 gene segment cluster led to an increase in the numbers of thymocytes ranging from 65 to 236 percent at all checkpoints studied, raising the possibility that the inclusion of this locus might retard thymocyte development (If so, this phenotype was lost when D_H_ coding sequence was substituted for Dβ). However, as in the case of B cells bearing a single, normal D_H_ (20) T cells using one normal Dβ achieved completely normal numbers of CD8 and CD4 T cells in the spleen. Conversely, there was a two-thirds to three-quarters loss of CD8 and CD4 splenic T cell numbers in DβYTL mice, respectively.

We crossed these mice with Foxp3-GFP reporter mice to evaluate the Treg population. We found a similar reduction in absolute T cell numbers in the CD4^+^Foxp3^+^ T cell population (Figure 3B and Table 2). A similar reduction in the numbers of mature, recirculating bone marrow B cells had been observed when the sequence of D_H_ was altered (21, 22). Thus, for both T cells and B cells, a reduction to only one, normal D sequence led to normal lymphocyte numbers in the periphery, but alteration of D sequence decreased peripheral lymphocyte numbers.

**TABLE 2:** Total numbers, SEM, and statistical analysis for Foxp3^+^ T cells in WT, Dβ2KO, and DβYTL.

	**WT ± SEM**	**Dβ2KO ± SEM**	**DβYTL ± SEM**	**WT vs. Dβ2KO**	**WT vs. DβYTL**	**Dβ2KO vs. DβYTL**
Thymus	2.74E + 05 ± 3.68E + 04	7.66E + 05 ± 5.65E + 04	1.52E + 05 ± 2.59E + 04	<0.001	0.06	<0.001
Spleen	1.07E + 06 ± 8.40E + 04	1.02E + 06 ± 1.08E + 05	4.32E + 05 ± 7.90E + 04	0.73	0.003	0.006
MLN	1.85E + 05 ± 3.77E + 04	2.17E + 05 ± 6.97E + 04	8.47E + 04 ± 8.94E + 03	0.67	0.12	0.04
PP	1.25E + 05 ± 1.01E + 04	1.52E + 05 ± 1.79E + 04	1.16E + 05 ± 2.86E + 04	0.39	0.75	0.25
Small Intestine	2.73E + 06 ± 4.91E + 05	3.20E + 06 ± 3.03E + 05	1.13E + 06 ± 8.64E + 04	0.58	0.008	0.006
Large Intestine	2.14E + 05 ± 1.13E + 04	1.86E + 05 ± 3.42E + 04	1.81E + 05 ± 5.90E + 04	0.65	0.57	0.93

### Alteration of Dβ Sequence Alters CDR-B3 Sequence

To test whether the sequence of Dβ could dictate CDR-B3 sequence signatures in conventional thymocytes and T cells, we performed RT-PCR of TCRβ transcripts obtained from the key quality control checkpoints (Figure 3A). For example, the transition from DN3a to DN3bc thymocytes is associated with β selection (23), the transition from thymocyte DP to single positive (SP) CD4 or CD8 T cells is associated with positive selection for recognition of self followed by negative selection to avoid pathogenic self-reactivity (24, 25). The surviving SP CD4 or CD8 thymocytes then egress the thymus, circulate, and enter lymphoid tissues such as the spleen (26). We focused our analysis on transcripts using a single commonly used Vβ, Vβ13-1

**FIGURE 4:**
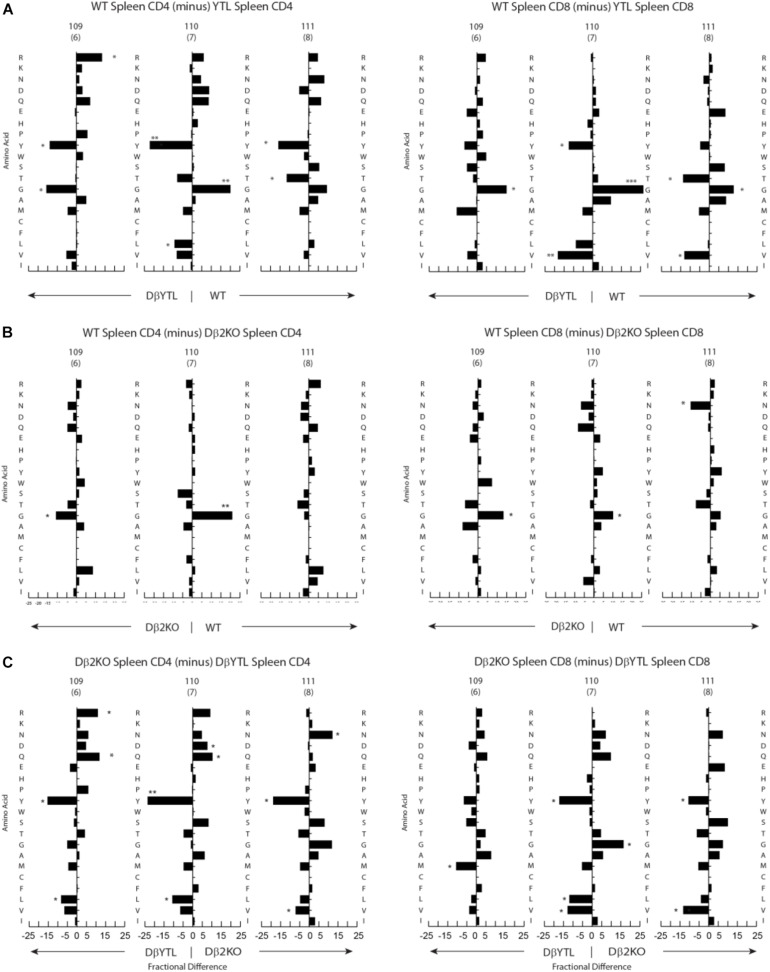
Percentage differences in amino acid usage in CDR-B3 among selected stages in T cell development. **(A)** WT splenic CD4 (left) and WT splenic CD8 (right) T cells versus DβYTL. **(B)** WT splenic CD4 (left) and WT splenic CD8 (right) T cells versus Dβ2KO. **(C)** Dβ2KO splenic CD4 (left) and Dβ2KO splenic CD8 (right) T cells versus DβYTL. Amino acids are in order from top to bottom by the Kyte-Doolittle hydrophobicity scale as modified by Eisenberg (9, 10) with charged amino acids [R (arginine), K (lysine), N (asparagine), D (aspartic acid), Q (glutamine), E (glutamic acid), H (histidine)] followed by neutral amino acids [P (proline), Y (tyrosine), W (tryptophan), S (serine), T (threonine), G (glycine)] then by hydrophobic amino acids [A (alanine), M (methionine), C (cysteine), F (phenylalanine), L (leucine), V (valine), I (isoleucine)], (*p < 0.05; **p < 0.01).

Using the IMGT numbering system, which places the conserved cysteine at the end of framework region 3 at position 104, we analyzed the positional representation of individual amino acids by percentage at positions 109–111 (Figure 4). This stretch of amino acids is positioned at the center of CDR-B3 where the residues are likely to interact with the peptide displayed on the MHC surface. Among all the T cell developmental stages studied in WT mice, glycine was typically the most common amino acid at all three of these positions. Threonine was also commonly used, while tyrosine and leucine were uncommon. The amino acid distribution at the central 109–111 positions during development among cells using the Dβ2KO control was similar, although not identical, to WT.

**FIGURE 5:**
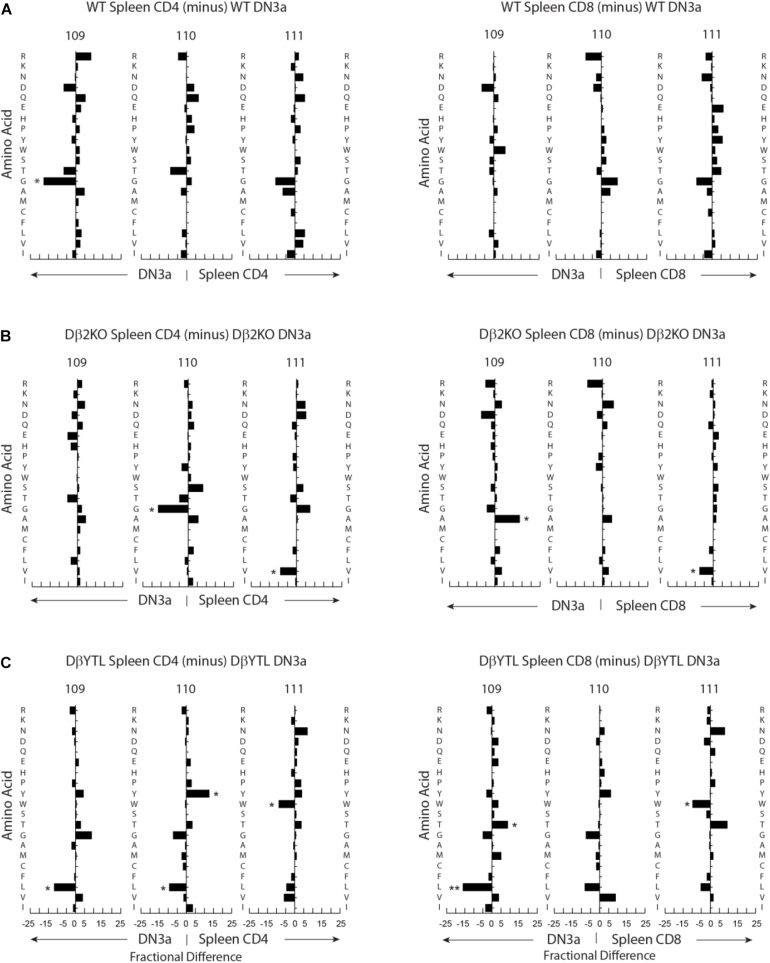
Fractional differences in amino acid usage in CDR-B3 109–111 among selected stages in T cell development. **(A)** WT splenic CD4 (left) and WT splenic CD8 (right) T cells versus WT DN3a thymocytes. **(B)** Dβ2KO splenic CD4 (left) and Dβ2KO splenic CD8 (right) T cells versus Dβ2KO DN3a thymocytes. **(C)** Dβ2KO splenic CD4 (left) and Dβ2KO splenic CD8 (right) T cells versus DβYTL splenic CD4 and CD8. The order of amino acids and the symbols used to report *p*-values are as given in the legend to Figure 4.

Changing the coding sequence of Dβ to DβYTL led to massive changes in the global amino acid composition of CDR-B3 (Figure 4C and Figure 5) in general, and at positions 109–111, in particular. At the DN3a stage, which is prior to β selection, use of the three reading frames was nearly equivalent (Supplementary Figure 2). At the amino acid level, DβYTL led to an increase in the use of tyrosine, threonine and leucine when compared to controls (Figure 5). Valine, which is encoded by RF2, was also common. Thus, just as in developing B cells, the germline sequence of the D appears to predetermine the distribution of amino acids used in CDR-B3. When amino acid representation at positions 109–111 in both WT and deletion control was compared as a function of development, no major differences were observed from the DN3a stage to the mature T cell stage. Thus, checkpoint passage did not appear to result in major changes in amino acid representation (Figure 5). However, when comparing mature DβYTL splenic T cells to DN3a DβYTL thymocytes, selection against leucine was readily apparent. In contrast, tolerance for tyrosine and threonine was observed (Figure 5).

### Positional Selection Against Leucine but Not Valine

**FIGURE 6:**
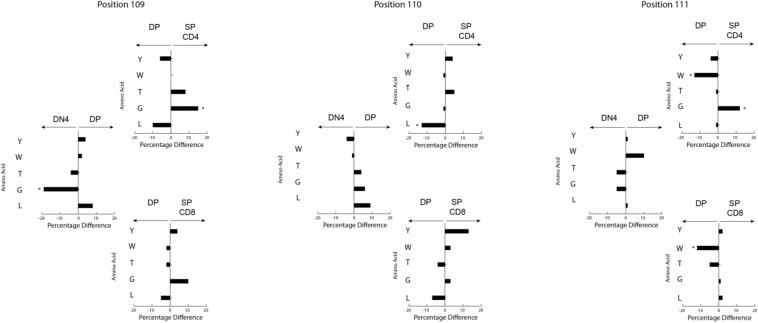
Loss of RF3 amino acids occurs at the thymic DP to SP checkpoint. Difference in selected amino acids between development stages (left to right) at CDR3 positions 109, 110, and 111 (top to bottom) in DβYTL mice, (*p < 0.05).

Stadinski et al. (27) have shown that changes at position 109 and 110 (positions 6 and 7 in their manuscript) from neutral to hydrophobic amino acids can elicit an autoreactive response. Here we find that at the same positions across all development there is a reduction in leucine and tryptophan but not valine (Figure 5). Because a reduction in tryptophan was not observed in either WT or Dβ2KO mice, we favor the view that reduction of tryptophan is more a bystander effect of being surrounded by leucine. We then looked only at amino acids that significantly changed during development from the DN3a stage to the mature stage at positions 109–111. We found that the reduction in leucine and tryptophan occurs in the thymus during the transition from DP/thymic SP transition double positive to single positive T cells (Figure 6). This reduction coincides with a decrease in the use of RF3 (Supplementary Figure 2), and with it a reduction in a specific set of “public” or semi-“public” CDR-B3s. This finding supports the view that selection against leucine is due to the global antigen binding characteristics of a TCR-B3 enriched for this hydrophobic amino acid, since the decline in its representation occurred at stages where the antigen specificity of the TCR is normally tested.

### Altered “Public” CDR-B3 Motifs

**FIGURE 7:**
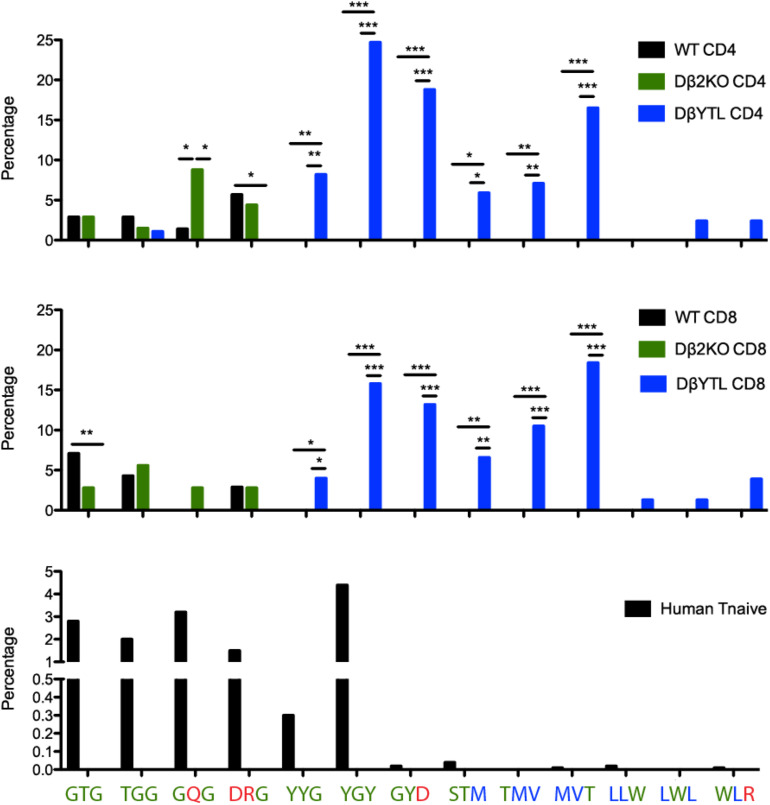
“Holes” present in the WT repertoire are filled in the DβYTL mice. These “holes” are present in humans, as well. A comparison of tripeptide usage in WT, Dβ2KO, and DβYTL mice in splenic CD4 (top panel) and CD8 (middle panel) T cells is shown, as well as tripeptide usage in human naïve T cells (bottom panel), (*p < 0.05; **p < 0.01; ***p < 0.001).

Comparisons of TCR repertoires have led to the recognition that some CDR-B3 sequences are common, or “public” between individuals; and others are rare, or “private” (28). Examination of these “public” sequences indicates that many include runs of germline sequence with more limited N addition (29). To test the effect of changing Dβ sequence on more “public” CDR-B3 motifs, we evaluated the frequency of triplet amino acids in mature splenic CD4 and CD8 T cells. For example, the coding sequence of Dβ1 yields four triplet peptide sequences (GTG, TGG, GQG, and DRG); whereas the coding sequence of DβYTL yields nine triplet peptide sequences (YYG, YGY, GYD, STM, TMV, MVT, LLW, LWL, and WLR) (Figure 7).

The change in representation of these peptide triplets was enormous and revealed unevenness, or even “holes,” in the repertoire. Triplets common in WT and Dβ2KO were not detected in our sampling of the DβYTL repertoire. Conversely triplets common in DβYTL were rare in WT and Dβ2KO. Although in WT and Dβ2KO the prevalence of triplet sequences was similar; the representation of tripeptides from the third reading frame of DβYTL T cells was reduced to WT and Dβ2KO levels. The only RF3 DβYTL tripeptide present in significant amounts was the one with the least amount of leucines.

Given the nucleotide sequence identity of Dβ1 in mouse and human, we evaluated the human repertoire for the prevalence of the same peptide triplets. Using previous high throughput sequencing data (30), we examined triplet sequences from various human T cell subsets and found that the unevenness in the repertoire extended to human (Figure 7). Nearly all triplets associated with use of DβYTL were rarely found in human sequences. The single exception was YGY. This peptide triplet is found in human Jβ2-2; and all instances of YGY in these human sequences were found at the 3′ end of the CDR3 where it is less likely to bind peptide.

### Alteration of Dβ Impairs the T Cell Response to Ovalbumin

**FIGURE 8:**
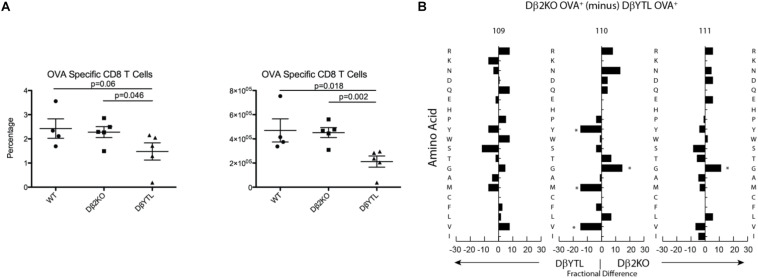
Alterations to Dβ sequence lower recognition of an ovalbumin immunodominant epitope. **(A)** Total cell number and percentage of OVA^+^ CD8 T cells. **(B)** Fractional difference in amino acid usage in CDR-B3 109–111 between Dβ2KO and DβYTL OVA^+^ CD8 T cells. Figure is representative data from two experiments with five individual mice per group, (*p < 0.05).

As a test of whether the categorical changes in CDR-B3 content created by replacing a Dβ with a D_H_ might alter TCR function, we immunized the three strains of mice with ovalbumin. After 7 days, we used an OVA-specific tetramer to measure the CD8 T cell response to the immunodominant epitope. Both by percentage and by absolute numbers, the response to this epitope was reduced in the DβYTL mice (Figure 8A). We sequenced the TCRβ of T cells that recognized OVA peptide and performed a positional CDR-B3 analysis (Figure 8B). The DβYTL OVA^+^ repertoire remained enriched for tyrosine and valine, and depleted of leucine, demonstrating that T cells with “non-preferred” amino acids profiles can create functional TCRs. However, as a population, T cells drawing from the altered Dβ demonstrated a lower ability, by percentage and absolute numbers, to recognize and respond to normally immunodominant epitopes.

## Discussion

Although the process of generating the antigen receptor repertoires that underlie adaptive immunity appears, at first glance, to be highly inefficient; our findings support the view that the sequence of D gene segments has undergone natural selection to counter the effect of random N addition by constraining the core of the antigen binding site repertoire to favor the presence of specific amino acids at specific positions. For both the WT and the Dβ2KO CD4 and CD8 repertoires, there are minimal differences in the representation of amino acids at specific positions in CDR-B3 between the DN3a stage and mature splenic T cells for both cell types (Figure 5). Thus, the mechanisms of somatic selection in the thymus and periphery appear to have a minor effect on the “core” repertoire; the T cell will use whatever D coding sequence is it supplied with. However, when the constraints that are normally imposed by natural selection of D gene sequence are violated, “normalcy” can only partially be attained by somatic selection, and then only for selected amino acids.

At the positive selection checkpoint, there was also a pruning of leucine from CDR-B3. Tyrosines, threonines, and valines, which are poorly represented in the WT repertoire, appear to pass through this selection step relatively unimpeded. Further studies should give us insight into whether there are certain global antigen “sensing sites” that can determine optimal amino acids in the CDR3, much like the role of the surrogate light chain in developing B cells (12). Still, stepping outside of the normal pattern of CDR-B3 amino acid content results in lower efficiency of T cell production and, in at least one case, lower efficiency in a response to antigen challenge (Figure 8). The extent to which these changes will affect responses to pathogens and self-antigens remains to be determined. However, should the parallels with immunoglobulin continue to hold true, we predict that susceptibility to infection and autoreactivity will also increase as a result of violating normal constraints on D coding sequence.

We found evidence of unevenness in the diversity of the T cell receptor repertoire, including potential “holes.” By tripeptide, the WT/Dβ2KO and the DβYTL are two distinct repertoires. A consequence of this is the loss of public TCR sequences. By comparing our sequences to known public and relatively public sequences (29), we found that roughly 2% of the WT and Dβ2KO sequences matched. However, we could not find any public sequences in the DβYTL sequences (data not shown). Since public sequences are highly germline with few N additions, it is possible that DβYTL mice lack the normal public D-containing component of the repertoire (Public sequences lacking D sequence are likely not affected). More sequencing would need to be done to confirm this possibility, but DβYTL-type mice could potentially be used as a means to test for the role of public, germline D-containing repertoires in immune responses.

Our findings support the view that “preferred” or even locally “optimal” preimmune repertoires exist and are beneficial to the immune function of the individual. The precise mechanisms by which these “preferred” repertoires enhance immune function remain to be determined. However, there are hints from the properties of specific amino acids where preference is observed.

In both controls and DβYTL, a preference for glycine was seen throughout T cell development. The bias toward use of glycine was previously attributed to a need for CDR-B3 flexibility (31), and our data does not argue against this point. A key difference between TCR and Ig is the enhanced use of tyrosine in CDR-H3, when compared to CDR-B3. If glycine favors flexibility, then it could have been argued that tyrosine would be selected against because its size might reduce flexibility. However, when replaced with a tyrosine (with a bulky side chain group that contains an aromatic ring and a hydroxyl group) there is no evidence of negative selection against this amino acid in the thymus. Instead, there was evidence of positive selection for tyrosine in SP thymocytes and even in splenic CD4 and CD8 T cells. Therefore, we believe that flexibility cannot fully account for the bias toward glycine in TCR.

Based upon our previous studies in immunoglobulin, we would propose an alternative explanation that changes in the physicochemical properties of the repertoire as a result of shifting biases in CDR-B3 amino acid content will influence patterns of epitope recognition and binding, and thus signaling. Although we would note that biases for flexibility and epitope binding patterns are not mutually exclusive.

One result of using D_H_ sequence in the TCR β locus was to make many TCR CDR-B3s more Ig CDR-H3-like (Figure 2) in their disdain for leucine and their preference for tyrosine as a function of checkpoint passage. The under representation of leucine is compatible with the recent finding that categorical use of hydrophobic CDR-B3 sequence increases the likelihood of self-reactivity (27). It seems likely that the preference for tyrosine is also due to the effect of this amino acid on antigen binding.

The affinity for antigen is higher in BCR than in TCR (32–34) and therefore creates longer lasting bonds with antigen, because the dissociation rate of binding is negatively correlated with affinity (35). We found that alterations in immunoglobulin to be more charged gave the BCRs a low affinity promiscuous phenotype (36). This is similar to T cells, where charged amino acids are more tolerated (Figure 1D and Figure 2). We posit that one of the reasons why tyrosine was not selected for evolutionarily is that glycine and charged amino acids may enhance the ability of the TCR to act as a sensor, because low affinity binding to peptide:MHC allows the TCR to sample a broader portion of the antigen milieu. In the presence of tyrosine, the balance between peptide:MHC promiscuity and specificity might be altered during T cell development, thus explaining the altered response to ovalbumin in our mutated mice.

We found that the non-random pattern of individual amino acid usage in CDR-H3 does not reflect physical inability for the T cell to create a TCR with these amino acids. If the amino acid is encoded in Dβ, the thymocyte will use it. Selection against particular amino acids appears to occur when antigen recognition is tested. The constraints imposed by the conservation of the Dβ create public or semi-public “holes” in the repertoire. The extent to which these holes create a normal physiologic immune deficiency against some antigens, including pathogens, has also yet to be determined, but it appears plausible that the unevenness of the repertoire could help explain why some epitopes, and even whole antigens, are more immunogenic than others.

In 2008, Cohn (37) pointed out two key facts about D gene segments. First, with occasional exceptions, D gene segments have been maintained throughout evolution as separate from V and J gene segments in both the immunoglobulin H chain locus and the TCR β chain locus. Second, D gene segments can be incorporated into both immunoglobulin and TCR in three different reading frames, each encoding a different peptide sequence, but whereas all three D reading frames are used in functional αβTCR, immunoglobulins exhibit a preference for only one of the three reading frames, wasting up to two thirds of all newly generated preB cells. In his view, these facts raised two questions. First, if a preferred reading frame is so important to immunoglobulins, why is there not a similar preference for a preferred reading frame in αβTCR and, second, why are D kept throughout evolution as separate gene segments?

Combined with our past work on immunoglobulin D and CDR-H3 (5) and that of others [e.g., (27, 31)], the present studies offer potential answers to these two questions. First, each D reading frame has a characteristic amino acid signature, which is conserved across evolution to a lesser (immunoglobulin) or greater (TCR) extent. In immunoglobulin, the preferred reading frame is neutral in hydrophobicity and enriched for tyrosine. Use of reading frames with hydrophobic amino acids, especially leucine, or charged amino acids, especially arginine, is discouraged both at the time of VDJ rearrangement and during passage through central and peripheral checkpoints. In contrast to the dramatic differences in amino acid content between immunoglobulin D reading frames, TCR Dβ are enriched for glycine in all three reading frames and lack tyrosine. This may explain the lack of preference for any one Dβ reading frame since they are all very similar to each other. Second, by allowing variation in the position of the D component in the final VDJ rearrangement due to varying sites of recombination and the effects of N addition, keeping the D separate from V and J enables individual amino acids encoded by the D to be put in a variety of places within the structure of CDR3, increasing the flexibility of the repertoire to generate a broad range of functional antigen binding sites.

In summary, our studies suggest that D gene segments were introduced and have evolved to facilitate the production of a broad range of “preferred” antigen binding sites while limiting the production of “suboptimal” or hazardous antigen binding sites. Thus, it is D for diversity, but also D for delimit or direct.

## Data Availability Statement

All data are included in the manuscript and in Supplementary Files.

## Ethics Statement

All animal experiments were approved by the University of Alabama at Birmingham (UAB) Institutional Animal Care and Use Committee.

## Author Contributions

ML took the lead role in performing the FACS analysis of T cell subsets, sequencing of the thymocyte and T cell subsets, sequence analysis, performing the ovalbumin challenge, preparing of the figures, and writing the manuscript. MK helped to analyze the data, prepared the figures, and wrote the manuscript. PB participated in the planning of the experiments, interpreted the data, and edited the manuscript. HS developed the concept of the project, directed the planning and execution of the studies, reviewed the data, and directed the writing of the manuscript. All authors contributed to the article and approved the submitted version.

## Conflict of Interest

The authors declare that the research was conducted in the absence of any commercial or financial relationships that could be construed as a potential conflict of interest.

## Supplementary Material

Supplementary Figure 1 Flow scheme for analysis and sorting of T cell subsets in the D altered and WT mice. **(A)** Flow scheme for DN thymocytes. DN thymocytes gated on live, lineage negative (CD3, CD4, CD8, CD11b, B220, and NK1.1) singlets **(B)** Flow scheme for DP and SP thymocytes. Gated on live singlets. **(C)** Flow scheme for CD4 and CD8 splenic T cells. Gated on live CD3^+^ singlets.

Supplementary Figure 2 Distribution of Dβ reading frame usage in selected thymocyte and splenic T cell subsets from WT and Dβ mutant mice.

Supplementary Table 1 Sequences of CDR-B3 from thymocyte and splenic T cell subsets from WT and Dβ mutant mice.
