# Supplementary figures and images for: Replacement of TCR Dβ With Immunoglobulin DH DSP2.3 Imposes a Tyrosine-Enriched TCR Repertoire and Adversely Affects T Cell Development
Source: Front Immunol. 2020 Sep 29;11:573413. doi: 10.3389/fimmu.2020.573413 (PMC7550431; doi:10.3389/fimmu.2020.573413)

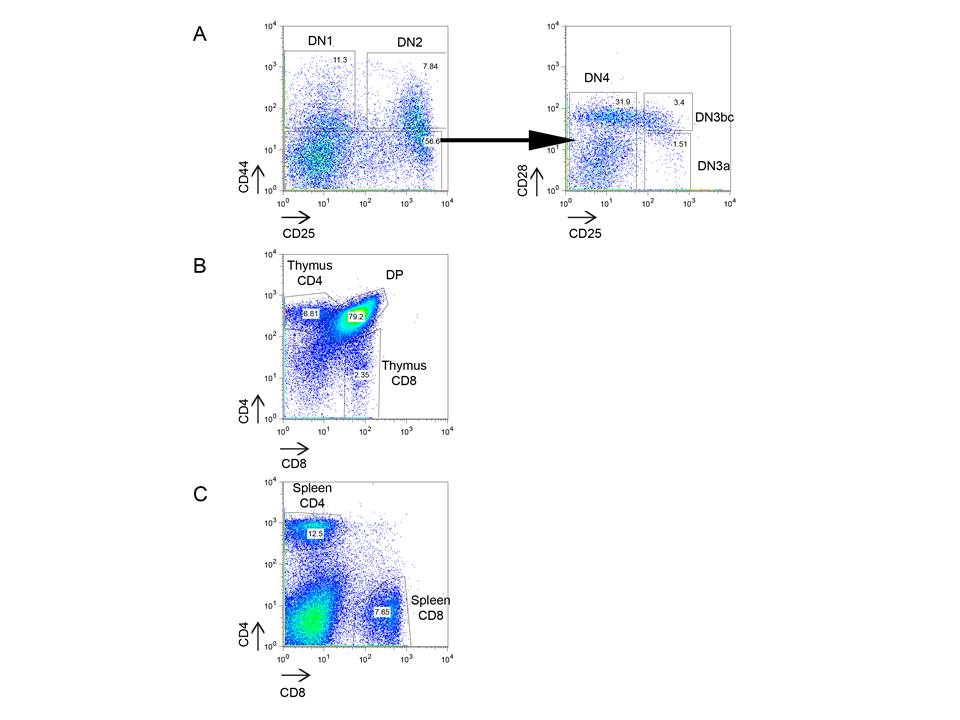

Supplement: Supplementary Figure 1 — Flow scheme for analysis and sorting of T cell subsets in the D altered and WT mice. (A) Flow scheme for DN thymocytes. DN thymocytes gated on live, lineage negative (CD3, CD4, CD8, CD11b, B220, and NK1.1) singlets (B) Flow scheme for DP and SP thymocytes. Gated on live singlets. (C) Flow scheme for CD4 and CD8 splenic T cells. Gated on live CD3+ singlets. [file Image_1.jpg]

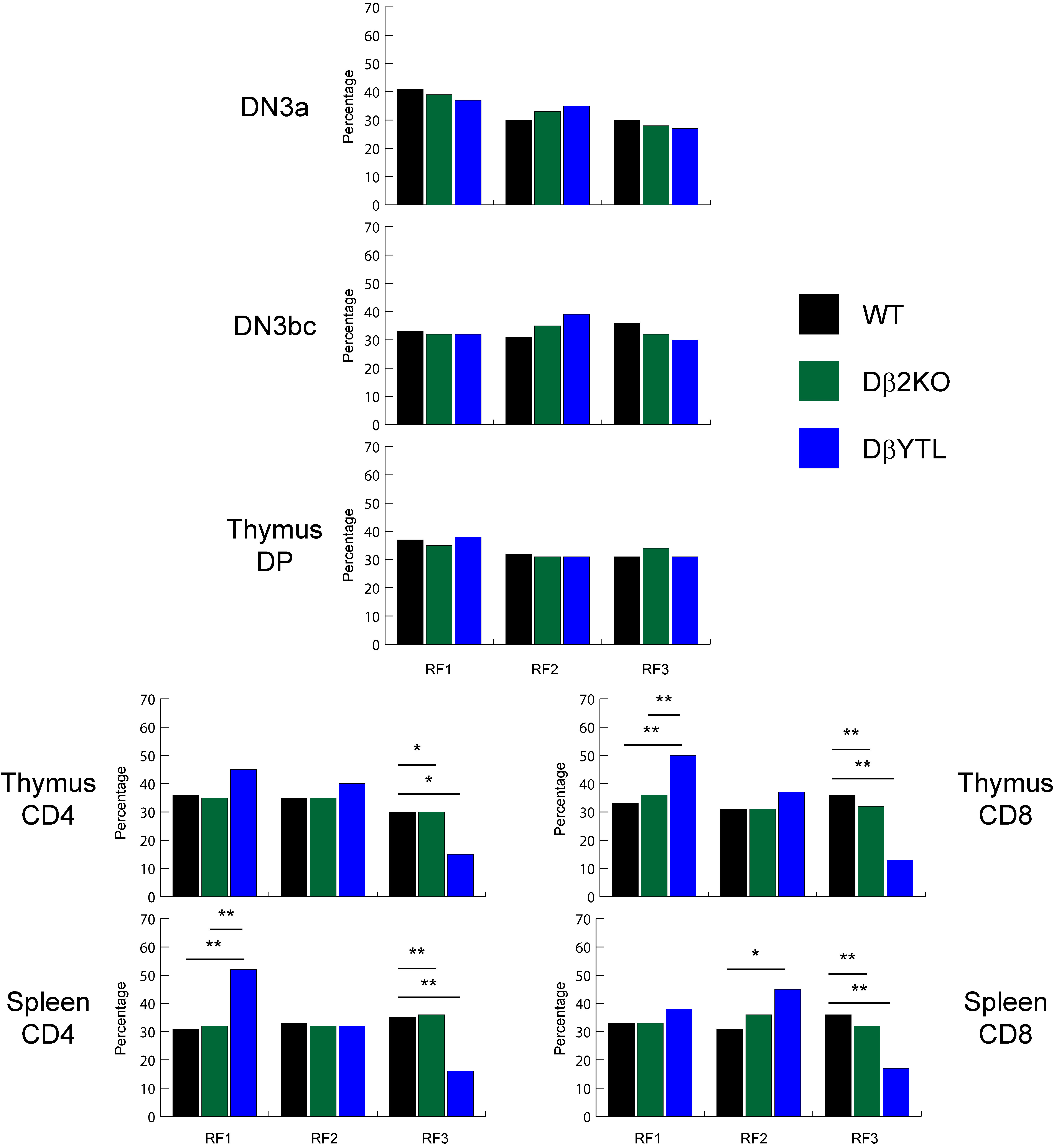

Supplement: Supplementary Figure 2 — Distribution of Dβ reading frame usage in selected thymocyte and splenic T cell subsets from WT and Dβ mutant mice. [file Image_2.jpg]
